# Robust Mutual Synchronization in Long Spin Hall Nano-oscillator Chains

**DOI:** 10.1021/acs.nanolett.3c02036

**Published:** 2023-07-14

**Authors:** Akash Kumar, Himanshu Fulara, Roman Khymyn, Artem Litvinenko, Mohammad Zahedinejad, Mona Rajabali, Xiaotian Zhao, Nilamani Behera, Afshin Houshang, Ahmad A. Awad, Johan Åkerman

**Affiliations:** † Physics Department, University of Gothenburg, 412 96 Gothenburg, Sweden; ‡ Center for Science and Innovation in Spintronics, Tohoku University, 2-1-1 Katahira, Aoba-ku, Sendai 980-8577, Japan; ¶ Research Institute of Electrical Communication, Tohoku University, 2-1-1 Katahira, Aoba-ku, Sendai 980-8577, Japan; § Department of Physics, Indian Institute of Technology Roorkee, Roorkee 247667, India; ∥ NanOsc AB, Kista 16440, Sweden

**Keywords:** Mutual synchronization, Spin Hall effect, Spin Hall nano-oscillators, Spintronic oscillators

## Abstract

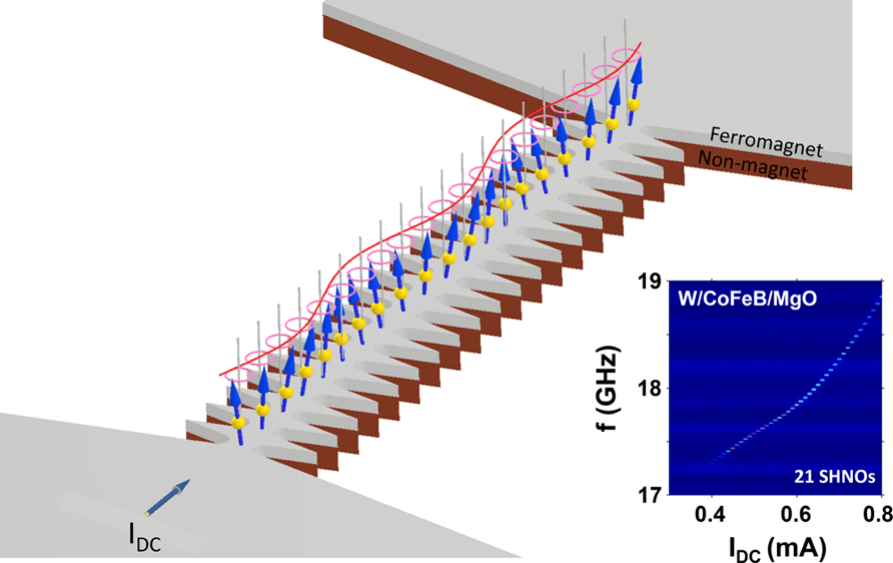

Mutual synchronization of *N* serially connected spintronic nano-oscillators boosts their coherence by *N* and peak power by *N*^2^. Increasing the number of synchronized nano-oscillators in chains holds significance for improved signal quality and emerging applications such as oscillator based unconventional computing. We successfully fabricate spin Hall nano-oscillator chains with up to 50 serially connected nanoconstrictions using W/NiFe, W/CoFeB/MgO, and NiFe/Pt stacks. Our experiments demonstrate robust and complete mutual synchronization of 21 nanoconstrictions at an operating frequency of 10 GHz, achieving line widths <134 kHz and quality factors >79,000. As the number of mutually synchronized oscillators increases, we observe a quadratic increase in peak power, resulting in 400-fold higher peak power in long chains compared to individual nanoconstrictions. While chains longer than 21 nanoconstrictions also achieve complete mutual synchronization, it is less robust, and their signal quality does not improve significantly, as they tend to break into partially synchronized states.

Since the advent of spin transfer torque driven magnetization precession in metallic spin valves,^1−4^ the interest in spintronic microwave oscillators has steadily increased.^5^ Mutual synchronization of these nonlinear microwave oscillators is of utmost importance for various applications such as efficient ultra-broadband signal generators,^6^ wireless communication, ultrafast microwave spectral analysis,^7,8^ and the recently developed interest in neuromorphic computing, among others.^9,10^ Moreover, researchers have recently demonstrated energy harvesting from wireless signals using synchronized oscillators in series.^11^ There have been many attempts to synchronize these oscillators over short and long ranges.^6,10,12−14^ However, the complex fabrication process of spin torque nano-oscillators (STNOs) raises a technological issue to scale their synchronization for high-frequency applications and hence the progress of synchronizing many STNOs has been rather slow.^6,11,14^

Thanks to the spin Hall effect,^15−17^ a new class of spintronic oscillators, known as spin Hall nano-oscillators (SHNOs), has emerged.^18−21^ Compared to STNOs, they rely on the current flowing in-plane, which makes their fabrication easier and allows for a much larger number of SHNOs to synchronize. In particular, nanoconstriction (NC) based SHNOs^18,19^ can be easily fabricated into 1D chains^22^ and 2D arrays.^23^ Earlier work has shown that up to nine SHNOs, separated by 300 nm, can be mutually synchronized to generate both higher output power and a narrower line width.^22^ Similarly, 2D arrays of up to 8 × 8 oscillators^23^ were found to synchronize completely. The number of synchronized oscillators along a dimension is hence limited to single digits (<10 oscillators).

In this work, we study mutual synchronization in much longer SHNO chains of up to 50 serially connected nanoconstrictions fabricated from W(5 nm)/CoFeB(1.4 nm)/MgO(2 nm),^24−27^ W(5 nm)/NiFe(3 nm),^28^ and NiFe(5 nm)/Pt(5 nm)^22,23^ material stacks (the order represents the actual stack sequence), focusing primarily on the W based SHNOs with their much lower threshold current and lower line width compared to Pt based systems (due to reduced spin pumping and lower inverse spin Hall effect). We find that robust and complete mutual synchronization can persist in chains of up to 21 oscillators, resulting in 1/*N* reduction in line width and *N*^2^ enhanced output peak power compared to single SHNOs. We also observe mutual synchronization in the longer chains but with deteriorated parameters, which we find to originate from a tendency for the longer chains to separate into shorter mutually synchronized sections.

Figure 1a shows the layout for a chain of nanoconstriction SHNOs made from a non-magnet (NM)–ferromagnet (FM) bilayer. The inset shows a scanning electron micrograph of an actual SHNO chain. A charge current flows in the film plane, and a magnetic field (H) is applied at an oblique OOP angle, θ. The spin Hall effect of the NM layer converts the charge current into a transverse spin current exerting an antidamping torque on the FM layer, which, above a certain threshold current, can generate auto-oscillations of the local magnetization in each nanoconstriction. These auto-oscillations are electrically detected via the anisotropic magnetoresistance (AMR). In this work, we explore 150 nm wide nanoconstrictions with a 200 nm center-to-center separation (the reduced separation, compared to our earlier work,^22^ increases the coupling strength^29^) and chain lengths of up to 50 nanoconstrictions. We primarily study and compare chains made from W/CoFeB/MgO and W/NiFe material stacks, where W was chosen for its very large spin Hall angle (θ_SH_ = −0.44; for details, see section S1 in Supporting Information) and the FM layers for their low damping of α_CoFeB_ = 0.025 and α_NiFe_ = 0.032. We also compare our results with 21 synchronized nanoconstrictions in the widely studied NiFe/Pt^22,23^ system (shown in the Supporting Information, section S2), where a much larger charge current density is required because of the lower spin Hall angle of Pt thin films. Figure 1b shows how the resistance of the SHNO chains increases linearly with the number of nanoconstrictions, with each nanoconstriction adding 105 and 264 ohm of resistance for W/NiFe and W/CoFeB/MgO, respectively. A schematic of the measurement setup is shown in Figure 1c. Further details about the measurements can be found in the Materials and Methods section.

**Figure 1:**
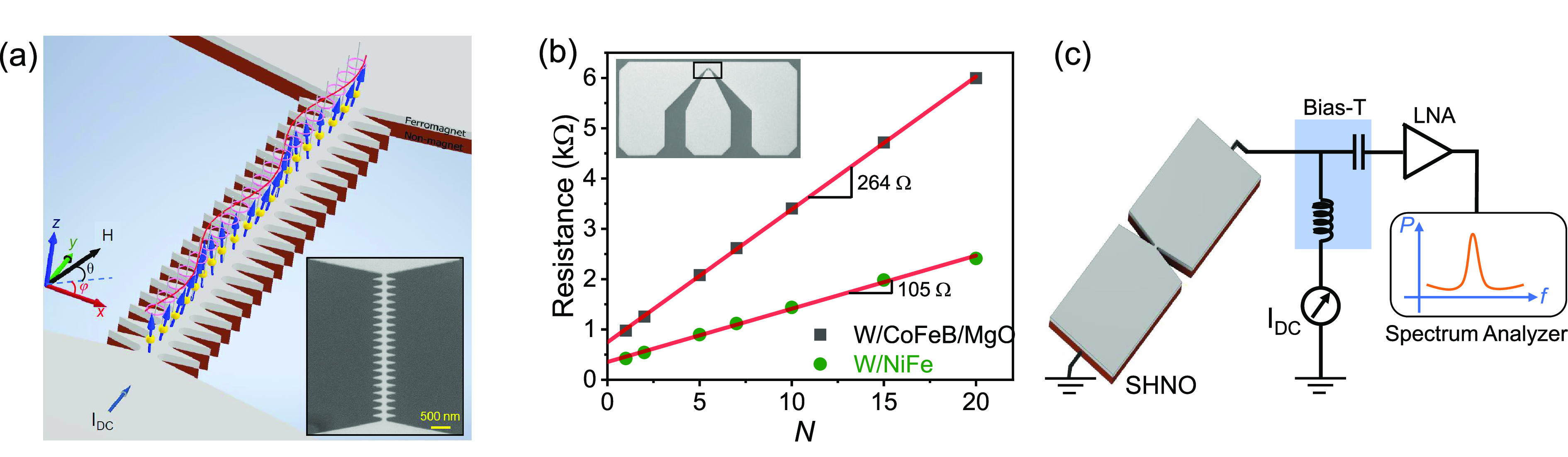
(a) Schematic representation of 21 nanoconstriction SHNOs in a chain fabricated from a non-magnet/ferromagnet bilayer. The inset shows a scanning electron microscopy image of an SHNO chain. (b) Resistance of the SHNO chains versus number of nanoconstrictions (*N*), showing the expected linear dependence. The inset shows an SEM image of the Ground-Signal-Ground pads connecting to the SHNO chain. (c) Schematic representation of the auto-oscillation measurement setup.

Figure 2 shows the power spectral density (PSD) of auto-oscillation in W/CoFeB/MgO (Figure 2a–f) and W/NiFe (Figure 2g–l) based SHNOs with *N* = 1–21. The behavior of chains with *N* ≥ 30 will be discussed in a later section. Both types of SHNOs show a positive nonlinearity, at given magnetic field magnitude and angles. The magnetic field direction and measurement conditions are optimized to attain positive nonlinearity (results for W/NiFe for weak in-plane fields are discussed in section S3, Supporting Information). As discussed in ref (24), the perpendicular magnetic anisotropy of the W/CoFeB/MgO stack increases its nonlinearity compared to W/NiFe, which is evident from Figure 2. The nonlinearity is also substantially higher for *N* ≥ 5 than for the single and double nanoconstrictions, which indicates spin waves (SWs) emission out of the nanoconstriction regions. For a small number of oscillators the energy losses by SW emission into the mesa are substantial,^30^ which limits the nonlinear frequency shift. Meanwhile, for larger *N*, the emitted waves contribute energy to the neighboring oscillators, increasing the nonlinearity.

**Figure 2:**
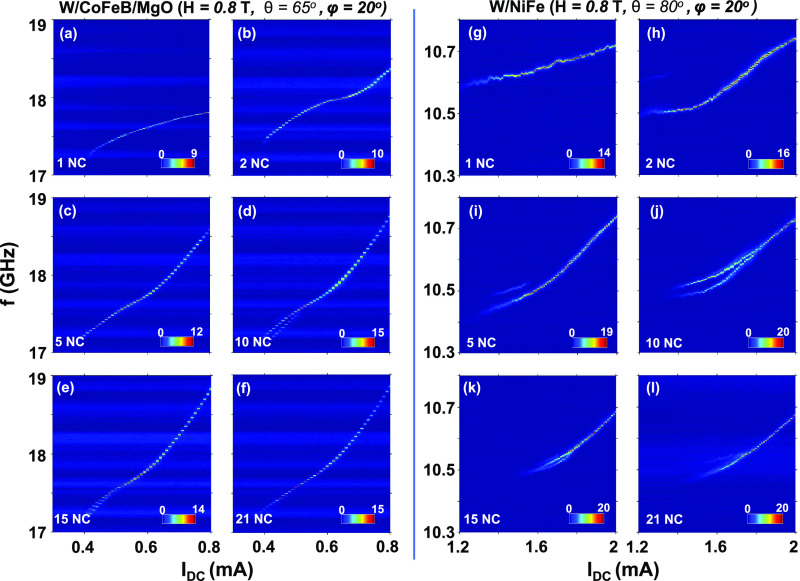
Power spectral density (PSD) of the microwave signal generated by a single nanoconstriction and up to 21 mutually synchronized nanoconstriction SHNOs for (a–f) W/CoFeB/MgO and (g–l) W/NiFe, respectively.

All chains show complete synchronization toward higher currents. The maximum peak power (indicated by the dB over noise scale) also increases with *N*. At lower currents, partial synchronization into primarily two or more separate signals can be clearly observed. The robust mutual synchronization is governed by a combination of dipolar- and spin wave-mediated coupling. The role of propagating spin waves and their comparison to dipolar coupling were first studied theoretically in nanocontact STNOs,^31^ where analytical calculations suggested a dominant role of propagating spin waves at separations larger than 100 nm. Spin wave beams were also responsible for robust synchronization of up to five nanocontact STNOs.^13^ Similar to nanocontact STNOs, nanoconstriction SHNOs share a common ferromagnetic layer, which suggests that the same arguments should apply. Following ref (31), for nanocontact STNOs the spin wave coupling strength should then be about twice that of dipolar coupling at a 200 nm separation (for typical auto-oscillation parameters). In the SHNOs, the constriction geometry allows much smaller direction for mode propagation compared to nanocontacts and hence will have even more dominated spin wave coupling strength.

The microwave signal is fitted with a single Lorentzian function to extract the power and line width. Figure 3 summarizes and compares the line width and peak power of 21 mutually synchronized oscillators for W/CoFeB/MgO and W/NiFe thin films; results for NiFe/Pt thin films are shown in the Supporting Information, section S2. The lowest line width of the W/CoFeB/MgO chain is 440 kHz (Figure 3a), with peak power just above 4000 nV^2^/Hz (Figure 3b). We observe the lowest line width of 134 kHz in the synchronized state of the W/NiFe based oscillator (Figure 3c), also with a peak power just above 4000 nV^2^/Hz (Figure 3d, measured for *I*_DC_ = 1.89 mA). The smaller line width of W/NiFe likely originates from both its weaker nonlinearity and its larger mode volume (thickness).^32^ For NiFe/Pt based SHNOs, we observe the lowest line width of 275 kHz with a much higher peak power of 40,000 nV^2^/Hz (see Supporting Information, section S2).

**Figure 3:**
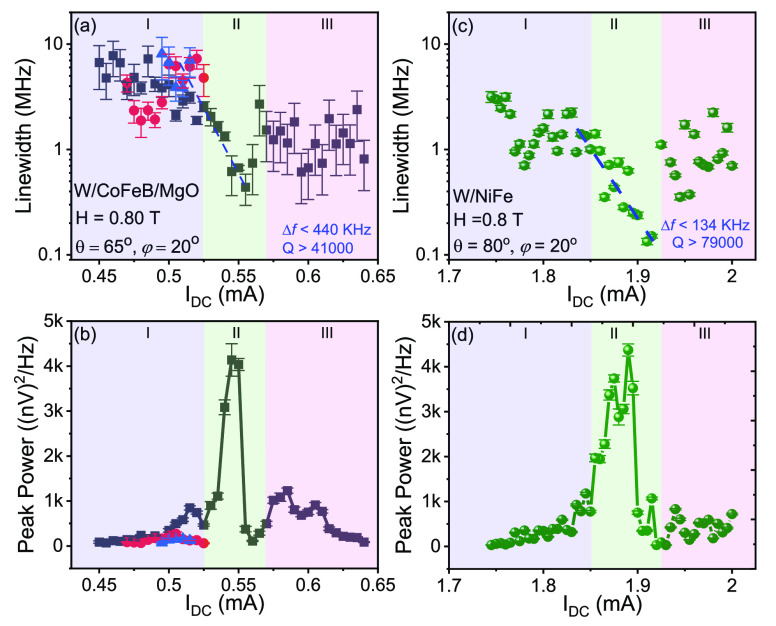
(a, b) Line width versus *I*_DC_ and peak power versus *I*_DC_ for 21 mutually synchronized W/CoFeB/MgO SHNOs. (c, d) Line width versus *I*_DC_ and peak power versus *I*_DC_ for 21 mutually synchronized W/NiFe SHNOs. The dashed blue line represents a complete mutual synchronization of 21 oscillators. The three color-coded regions represent the unsynchronized state (blue), the robustly synchronized state (olive), and the high-current, unstable synchronized state (pink).

However, once this highest-quality signal is achieved, increasing the current further deteriorates the signal quality to intermediate values. This deterioration does not seem to be related to a loss of mutual synchronization, as we only observe a single signal in all devices in this current range. Instead, this behavior coincides with the change in curvature described above, indicating that it could be due to a change in the auto-oscillating mode character. We hence define three different regions: (I) incomplete partial synchronization with relatively poor signal quality, (II) complete mutual synchronization with the best signal quality, and (III) a possible different auto-oscillating regime with intermediate signal quality.

Figure 4a and b show the variation of line width and peak power with the number of oscillators for both the W/CoFeB/MgO and W/NiFe systems. We observe a 1/*N* dependence for the spectral line width, in agreement with the oscillator synchronization theory, which depicts the enhancement of total mode volume. The peak power is found to follow a quadratic (*N*^2^) dependence, which is also consistent with the nonlinear oscillator theory.

**Figure 4:**
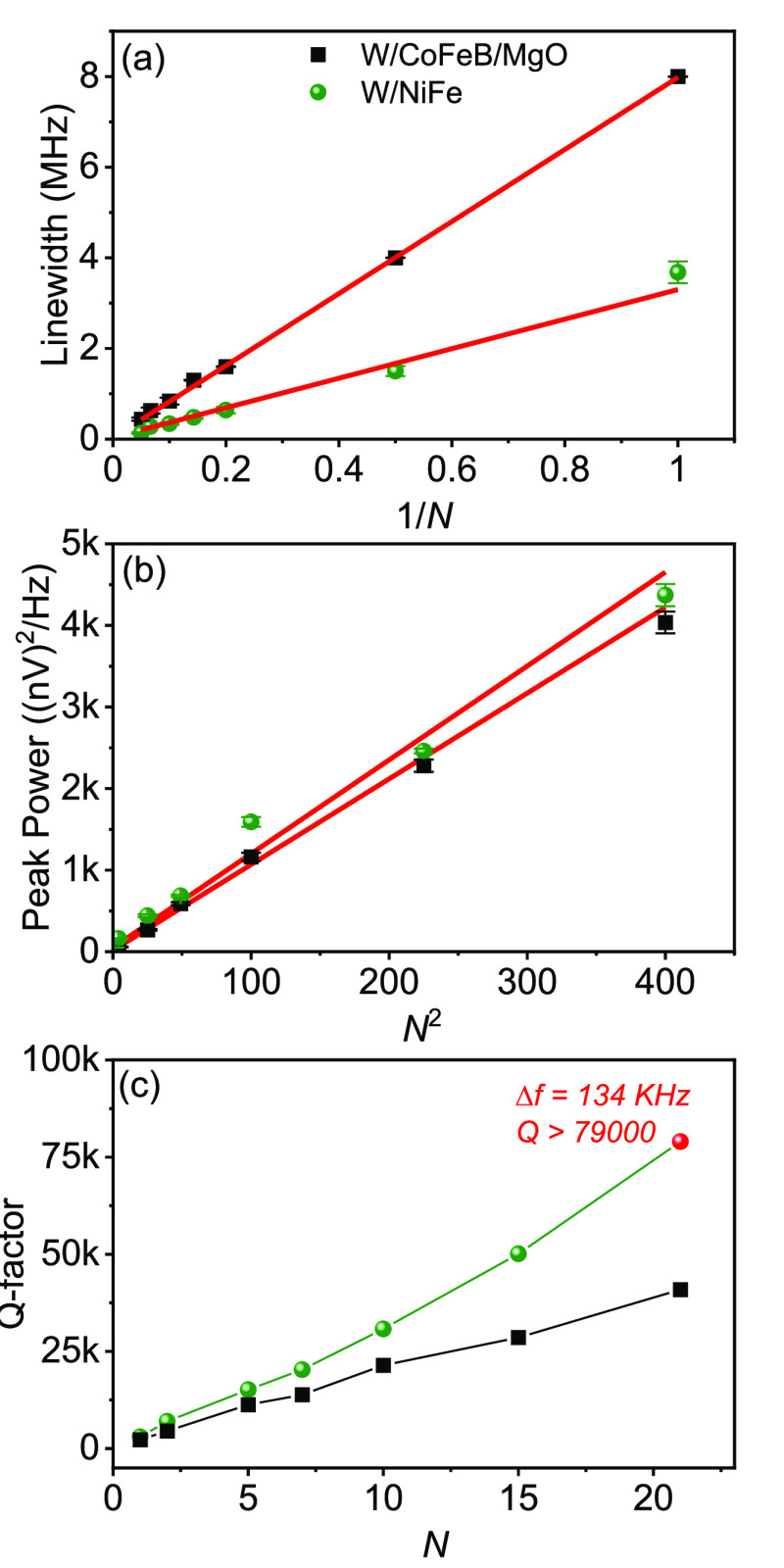
(a) Line width versus inverse of the number of nanoconstrictions (1/*N*) in a chain of SHNOs for W/CoFeB/MgO and W/NiFe based oscillators. (b) Peak power versus square of the number of oscillators (*N*^2^) for W/CoFeB/MgO and W/NiFe based oscillators. (c) *Q* factor of SHNOs with the number of oscillators (*N*). Black squares are for W/CoFeB/MgO and green circles represent W/NiFe. The red solid line represents the linear fit.

The combination of high auto-oscillation frequencies and low line widths leads to very high quality factors (*Q* = *f*/Δ*f*) for the synchronized chains. Figure 4c shows *Q* versus *N* for both the W/CoFeB/MgO and W/NiFe systems. We observe *Q* > 79,000 for 21 mutually synchronized SHNOs in W/NiFe thin films, which is the highest quality factor reported for oscillators in a single chain (which also results in higher output power). This is comparable to the earlier observed *Q* of 170,000 in two-dimensional NiFe/Pt arrays of 8 × 8 oscillators. For the W/CoFeB/MgO oscillators, we found a *Q* of 41,000, which is again the highest of any spintronic oscillator chains operating at frequencies higher than 15 GHz. For comparison with mutually synchronized oscillators based on magnetic tunnel junctions, recent demonstrations^6^ of 8 mutually synchronized magnetic tunnel junctions (MTJs) resulted in a *Q* factor of 7400. In Supporting Information section S4, we present a benchmarking of our oscillators compared to other spintronic oscillators and their synchronized systems. We find that the present reports have best-in-class *Q* factors with an optimum output power. To further increase their output power, one would add magnetic tunnel junction based readout.

To investigate synchronization beyond 21 SHNOs, we fabricated 30, 40, and 50 nanoconstrictions in series. It is noteworthy that we do observe single-frequency microwave signal generation also in these much longer oscillator chains. However, no further line width reduction nor any further increase in the output power was observed. This could be due to an increasing out-of phase synchronization of SHNOs in the longer chains, as illustrated by the spins in Figure 1a, if there is a small relative phase shift between individual nearest neighbor SHNOs that does not affect shorter chains but builds up to a reduced total output power in the longer chains. Figure 5 shows the PSD for 30, 40, and 50 nanoconstrictions in a chain for W/CoFeB/MgO thin films. For 30 nanoconstrictions in a chain (Figure 5a), we found the lowest line width of 800 kHz and a peak power of less than 400 nV^2^/Hz, which is 1 order of magnitude less than that of the robustly synchronized 21 SHNOs, as shown in Figure 4. Figure 5b shows the transition of partially synchronized microwave emissions into a single synchronized mode at different *I*_DC_ values. However, as observed in Figure 5b the spectra at 450 and 490 μA (partially synchronized states) show lower line widths and higher amplitude than those for fully synchronized states at 540, 640, or 730 μA current. This clearly shows that the partial synchronization of oscillators results in better spectral parameters than those for the full synchronization of oscillator chains with more than 30 nanoconstrictions. In other words, the longer chains may very well synchronize completely but be better described as weaker synchronization of partially synchronized subsections. This lack of robust synchronization for more than 21 nanoconstrictions may arise from statistically more fabrication defects in a larger ensemble or a temperature gradient in SHNO chains (between inner and outer SHNO due to different thermal sinks) or be due to larger Joule heating in longer chains, and/or result from the increase of an accumulative phase difference in the chains. To understand the effect of Joule heating and temperature gradient, we performed COMSOL simulations for varying numbers of oscillators (see Supporting Information, section S5). We found a temperature difference as large as 25 K between interior SHNOs and outer SHNOs in longer chains of W/NiFe. This is a significant temperature difference, and its impact on the intrinsic frequency of each nano-oscillator is likely substantial (previously observed by opto-thermal effects^33^). One possible solution to reduce the temperature gradient will be to use electrically insulating materials with large thermal conductivity, i.e. Al_2_O_3_ or SiC, as seed layers or encapsulation. This will significantly increase the thermal budget of the devices. While still remaining within the locking bandwidth, these larger differences in intrinsic frequencies likely reduce the coherence of the mutually synchronized state. Figures 5c and d show similar results for 40 and 50 nanoconstrictions in a chain, respectively. Though full synchronization in longer chains of more than 21 nanoconstrictions is not very robust, it still shows a clear interaction between oscillators (either in-phase or out-of-phase), which can be very useful for neuromorphic computing using a large number of spins (nanoconstrictions).

**Figure 5:**
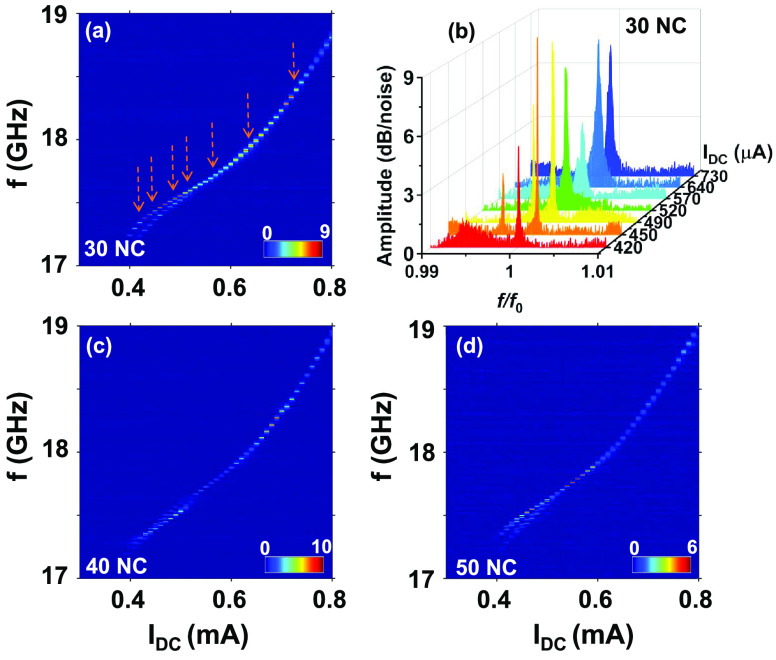
(a) Power spectral density for synchronized 30 nanoconstriction SHNOs in a chain. (b) Microwave spectrum for synchronized 30 nanoconstrictions in a chain for a few current values (*I*_DC_) marked with arrows in part (a). (c, d) Power spectral density for mutually synchronized (c) 40 nanoconstriction and (d) 50 nanoconstriction SHNOs in a chain for W/CoFeB/MgO thin films.

We have successfully demonstrated the robust mutual synchronization of a large number of SHNOs in a single chain. This observation leads to various applications that can be realized by using these oscillators. The lower line width and the significantly larger output power enable these oscillators for coherent frequency signal generation applications, as well as for wireless communications. Using the phase-locked loop method, the microwave oscillations in chains can be further stabilized, generating coherent oscillations that can be directly implemented in many microwave applications.^34^ The mutual synchronization of these oscillators in a chain can be explored for bioinspired computing and beyond^9,23,35^ where each oscillator behaves as a neuron. Combined with voltage^26,27,36^ and/or memristive^25^ control of synchronization (synaptic weights), these large chains can be used to locally or globally control the coupling between oscillators (neurons). The present work also serves as a stepping stone in the direction toward further scaling mutual synchronization to much larger square or rectangular arrays well beyond the previously demonstrated 64 oscillators. As these oscillator arrays can be fabricated in a tiny area, this enables the possibility of scaling/miniaturizing the neuromorphic networks based on these oscillators. The large frequency tunability with current also makes these oscillators ideal for ultrafast sweeping spectrum analysis, where neither vortex oscillators nor uniform MTJs can so far offer a wide resolution bandwidth.^7,8^ Moreover, larger output power over synchronization in long chains can be used for signal amplification^37^ and energy harvesting.^11^ With recent demonstration of energy-efficient spin Hall materials^38^ and the reduction of constrictions size,^21,39^ the required threshold current can be significantly reduced, allowing operation of these devices with ultralow power.

In summary, we show that robust in-phase mutual synchronization of nanoconstriction based SHNOs can be achieved in very long chains. Improved performances are achieved from the advanced fabrication process, large spin Hall effect of W thin films, and optimized separation between SHNOs giving rise to stronger coupling. The long-range synchronization not only shows an enhanced output power but also an improved line width of as low as 134 kHz for W/NiFe based heterostructures. The low-current and low-field operation of these oscillators along with their large frequency tunability, with both current and magnetic field, make them ideal for various emerging spintronic applications. In the longest chains, mutual synchronization is less effective in improving the microwave signal properties with evidence of partial or increasingly out-of-phase synchronization. These results not only enhance our understanding of the mutual synchronization of these oscillators but also pave the way toward making larger networks of these oscillators for neuromorphic computing applications.

## Materials and Methods

### Sample Fabrication

We utilized the well studied W(5 nm)/CoFeB(1.4 nm)/MgO (2 nm) and W(5 nm)/NiFe(3 nm) heterostructures for the fabrication of the microwave nanoconstriction SHNOs used in the experiments. The NM/FM structures were deposited using magnetron sputtering on a high-resistance intrinsic Si substrate (*ρ* > 10,000 *μ*ohm·cm) at room temperature. The sample stacks were capped with 4 nm Al_3_O_3_ thin films. The growth of thin films was carried out using an AJA Orion 8 sputtering system with a base pressure of 3 × 10^–8^ Torr. The samples were then coated with 40 nm of hydrogen silsesquioxane (HSQ) negative tone electron beam resist. The SHNO chains used in the experiments were then fabricated using a combination of e-beam lithography (Raith EBPG 5200) and Ar-ion etching: more details can be found in ref (26). The top contact pads are fabricated using laser writing-based direct lithography followed by deposition of Cu(800 nm)/Pt(20 nm) thin films for the Ground-Signal-Ground coplanar waveguide. In this experiment, we utilized SHNOs with 150 nm nanoconstriction width and 200 nm separation between the SHNOs in a chain.

### Experimental Setup

All of the measurements were performed at room temperature. The microwave measurements were performed using a custom-built probe station using GSG probes manufactured by GGB Industries. Figure 1c shows a schematic representation of the measurement setup. All measurements were carried out at a fixed IP angle with an OOP rotatable sample stage between the electromagnet poles at room temperature. Different OOP angles were used to generate positive nonlinearity in the system. To excite microwave emission, a positive DC current *I*_DC_ was applied to the devices through the inductive port of a bias-tee, while the microwave signal was detected using a high-frequency port. The resulting power spectral density (PSD) of the auto-oscillating signal (after amplification using a low-noise amplifier) was captured using a Rohde and Schwarz (10 Hz to 40 GHz) spectrum analyzer. The power spectral densities plotted in Figures 2 and 5 show the measured signal with a spectrum analyzer (dB/noise), and Figures 3 and 4 show the peak power (PP) calculated by subtracting the amplification gain and considering the impedance reflection correction (*Z*_C_). We utilized the following formula to extract the peak power from the measured signal.
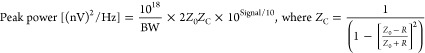
1Here, *Z*_0_, *R*, and BW are the impedance load (50 Ω), the resistance of the device under test (SHNO), and the resolution bandwidth of the spectrum analysis. Moreover, the Signal represents the RF signal measured in dBW for devices and can be calculated from the measured data as Signal = (measured signal in spectrum analyzer – LNA amplification gain – 30); subtraction of 30 converts the dBm to dBW.

## Supporting Information Available

S1: AMR and STFMR measurements of the W/CoFeB/MgO system. S2: Mutual synchronization in NiFe/Pt based 21 SHNOs in a chain. S3: Mutual synchronization at in-plane angles. S4: Benchmarking the quality factor versus output power. S5: Temperature gradient and increased Joule heating: COMSOL simulations. (PDF)
