# Supplementary material for: Robust Mutual Synchronization in Long Spin Hall Nano-oscillator Chains
Source: Nano Lett. 2023 Jul 14;23(14):6720–6. doi: 10.1021/acs.nanolett.3c02036 (PMC10375588; doi:10.1021/acs.nanolett.3c02036)
Supplement: Supplementary file 1 — nl3c02036_si_001.pdf [file nl3c02036_si_001.pdf]

Supplementary Information

# Robust Mutual Synchronization in Long Spin Hall Nano-Oscillator Chains

Akash Kumar,[*,†,‡,¶] Himanshu Fulara,[§] Roman Khymyn,[†] Artem Litvinenko,[†] Mohammad Zahedinejad,[‖] Mona Rajabali,[‖] Xiaotian Zhao,[†] Nilamani Behera,[†] Afshin Houshang,[†] Ahmad A. Awad,[†,‡,¶] and Johan Åkerman[*,†,‡,¶]

†*Physics Department, University of Gothenburg, 412 96 Gothenburg, Sweden.*

‡*Center for Science and Innovation in Spintronics, Tohoku University, 2-1-1 Katahira, Aoba-ku, Sendai 980-8577 Japan*

¶*Research Institute of Electrical Communication, Tohoku University, 2-1-1 Katahira, Aoba-ku, Sendai 980-8577 Japan*

§*Department of Physics, Indian Institute of Technology Roorkee, Roorkee 247667, India*

‖*NanOsc AB, Kista, Sweden.*

E-mail: akash.kumar@physics.gu.se; johan.akerman@physics.gu.se

## S1: AMR and STFMR measurements

Figure S1 summarizes the an-isotropic magnetoresistance (AMR) and spin-torque ferromagnetic resonance measurements for W/CoFeB/MgO based microstrip (4×14 $\mu m^2$) devices. Figure S1a shows frequency-dependent STFMR measurements for the trilayer heterostructures for a frequency range of 4-12 GHz measured at an in-plane angle of 20°. Figure S1b shows a scanning electron micrograph image of the fabricated devices with ground-signal-

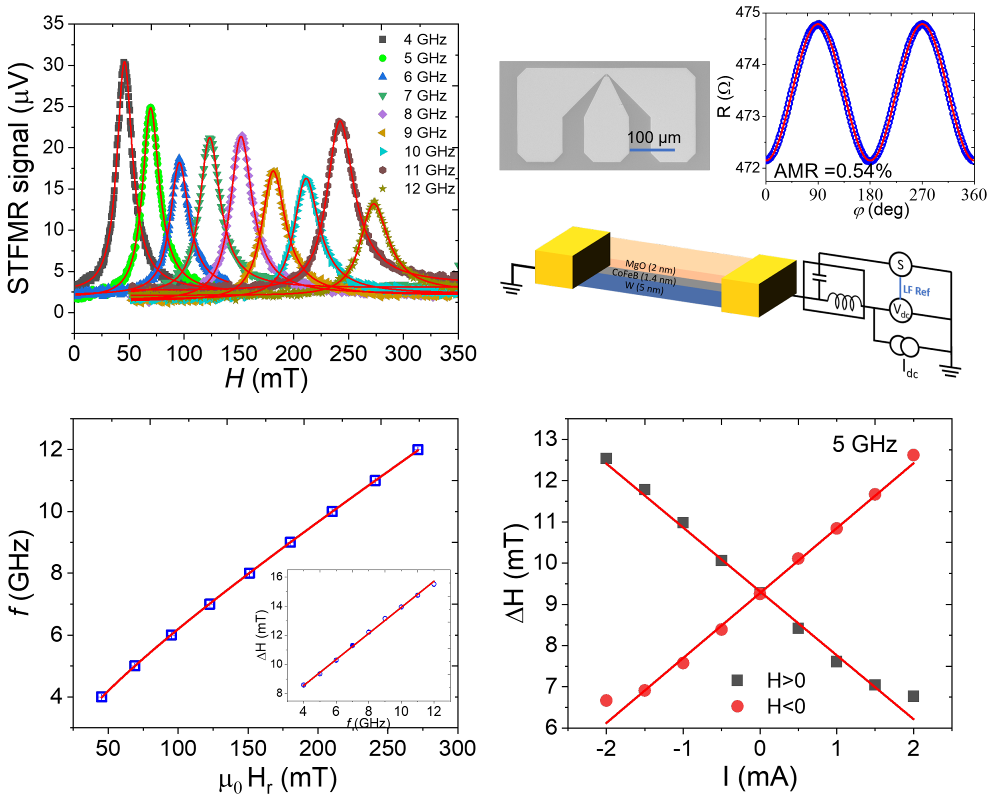


Figure S1: (a) Measured STFMR signal for W/CoFeB/MgO thin films, (b) measurement schematic and observed AMR response of the microstrips of $4{\times}14\ \mu\text{m}^2$. (c) The frequency versus resonance field. The effective saturation magnetization can be calculated by fitting the f versus Hr using Kittel equation[1]. The inset of (c) shows the linewidth versus frequency. (d) The modulation of linewidth versus applied DC current, used to calculate the spin Hall angle using linewidth modulation method[3].

group (GSG) contact pads. Figure S1b also show the measurement system and AMR signal observed for the device. We found a large AMR of about 0.54 %. Figure S1c shows the frequency versus resonance field data (solid lines show fitting with Kittle Formula[1]) and the inset show the linewidth versus frequency measured (the linear fit shown with solid lines is used to determine the Gilbert damping). We found a large linewidth modulation (see Fig. S1d) and spin Hall angle of -0.44 for W thin films, these values are in agreement with our previous results[2].

# S2: Mutual synchronization in NiFe/Pt based 21 SHNOs in a chain

Figure S2 shows the power spectral density of auto-oscillations and extracted parameters observed for 21 oscillators in chain with NiFe (5 nm)/Pt (5 nm) system. The lower spin Hall angle of Pt and larger thickness of the thin films requires a much larger charge current, but it also increases the output power due to increased AMR response from thicker NiFe thin film. It is evident from the PSD that we observe a single coherent peak with integrated output power of larger than 300 pW and peak power larger than 40,000 $nV^2/Hz$.

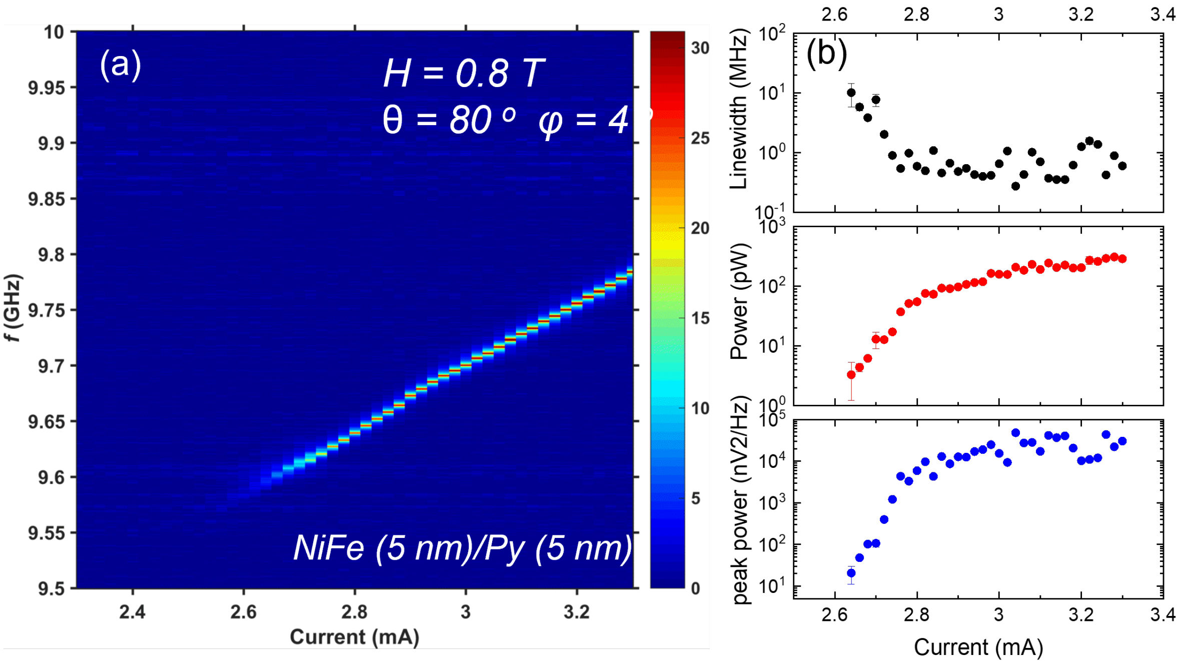


Figure S2: Power spectral density for NiFe/Pt based 21 nano-constriction SHNOs in a chain. The high AMR of thicker NiFe results in large output power.

# S3: Mutual synchronization at in-plane angles

The in-plane angle allows the operation of SHNOs at much lower magnetic fields but results in either zero non-linearity (localized modes) or negative non-linearity (bullet mode). The

negative non-linearity does not allow robust mutual synchronization and results in degraded power and linewidth, this could be understood because of the absence of propagating spin wave modes at lower magnetic fields, moreover, the dynamical edge/bullet modes in negative non-linearity do not allow easier coupling between SHNOs separated by large distances[4]. To further confirm the same in the present system, we have performed auto-oscillation measurements for the W/NiFe system at in-plane field angles ($\theta = 0°, \phi = 20°$). Figure S3 shows PSD for 1-21 NC in a chain with negative non-linearity. We observe that for negative non-linearity, not all oscillators synchronize and moreover the phase of oscillators does not allow a robust synchronization which results in deteriorated parameters.

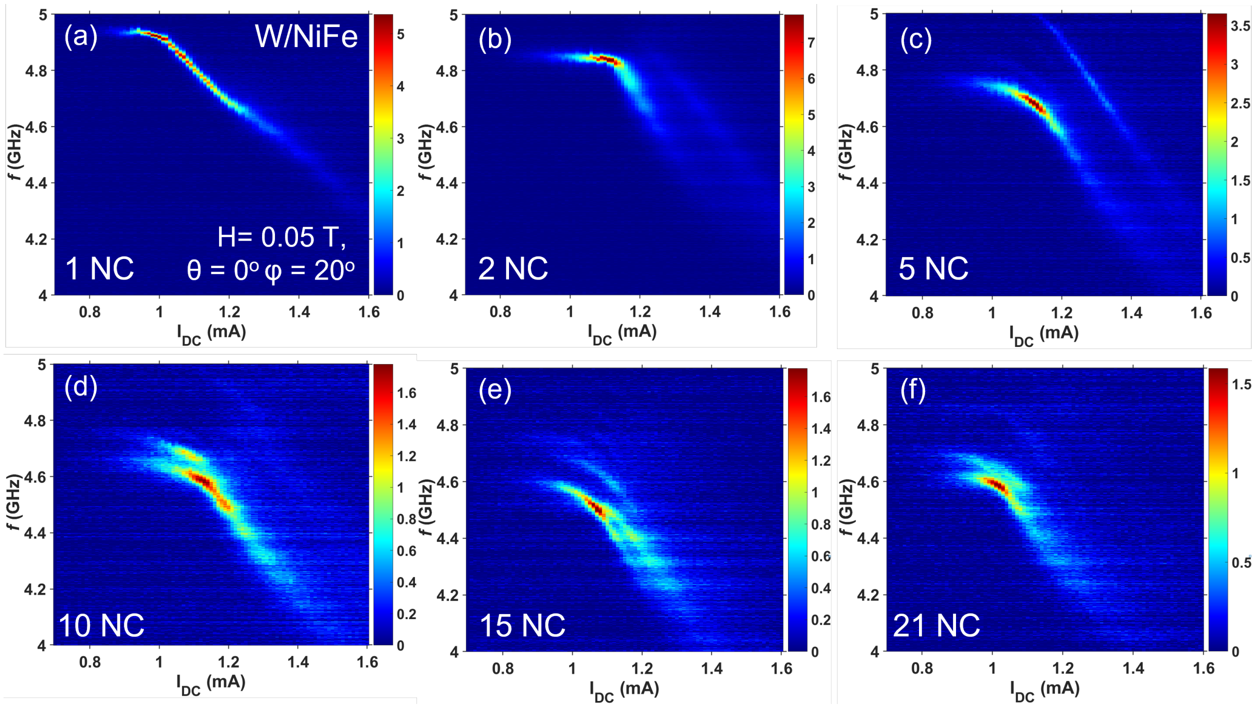


Figure S3: (a-f) Power spectral density at in-plane angle measurements for the microwave signal generated by a single nano-constriction and up to 21 nano-constriction SHNOs of W/NiFe thin films.

## S4: Benchmarking the quality factor versus output power

It is interesting to compare $Q$–factor (and output power) more extensively with the literature. In Fig. S4, we show results from a large number of previous works, expressed as $Q$–factor versus output power, for both single (hollow symbols) and synchronized (solid symbols) oscillators. Both here and further on, we use the abbreviations SHNOs: spin Hall nano-oscillators (shown with triangles), MTJs: nano-pillar magnetic tunnel junctions which include vortex oscillators (shown with circular symbols), and Nano Contacts: nano-contact spin valve structures (shown with rhombus). One can observe that the vortex oscillators[15–19] deliver the largest output power (most lie on the right side of the graph). However, vortex oscillators mostly operate at much lower RF frequencies (0.1 GHz to 1.5 GHz) and hence result in lower quality factors. Other MTJs and nano-contact devices have larger frequency tunability but have lower output power and/or higher operational linewidth resulting in

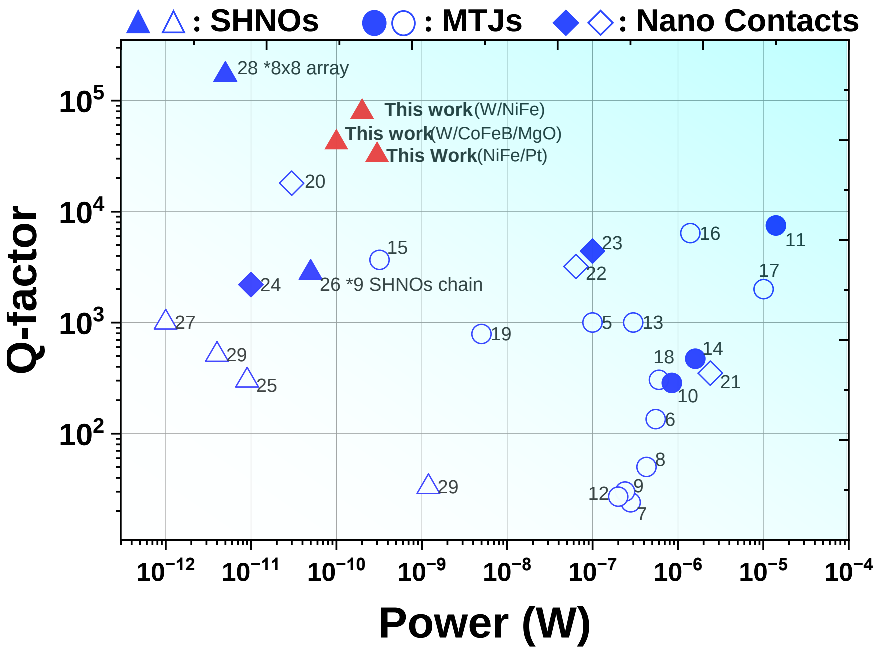


Figure S4: **Bench-marking of spintronic oscillators**: $Q$–factor versus integrated output power of various spintronic oscillators and their synchronized systems (shown with filled symbols). The data comprise the best performance nano-pillar MTJs[5–14], vortex MTJs[15–19], Nano-contact spin valves[20–24] and SHNOs[25–29].

poor performance as signal generator. The synchronized MTJs have shown good results and are even employed for power harvesting[10]. Though they also end up with large linewidth and hence low $Q$–factor. The best $Q$–factor found for nano-contact spin valve was about 18000[20], which resulted in poor output power. The recently introduced nano-constriction SHNOs have already shown great promise with their narrow linewidth and high frequency operation[30]. Even a single oscillator results $Q$–factor in the range of 2000-4000[25,27,31], though their output power is extremely low. In our previous work with 2D arrays of 64 oscillators, we observed an enormous $Q$–factor of 179,000, though in a white noise regime (measured at short time scales). In the present work, we have improved our output power and can reach upto 200 pW with sustaining a high $Q$–factor of >79000 (for W/NiFe thin films). The NiFe/Pt system shows even higher output power of 300 pW with Q $>$ 32,000. Further improvements in extending synchronization to rectangular arrays and fabricating tunneling magneto-resistance based readouts will bring these oscillators among the best-performing spintronic oscillators with large output power and high $Q$–factor.

## S5: Temperature gradient and increased Joule heating: COMSOL simulations

To understand the effect of temperature gradient and increased Joule heating in longer chains of SHNOs, we have performed a systematic COMSOL simulation for a varying number of oscillators in chain. We used COMSOL modules Electric Currents (ec) to simulate the current density variation in the nanoconstrictions together with the Heat Transfer in Solids (ht) module. Multiphysics simulations were performed using the Electromagnetic Heating (emh1) module. In our simulation, we take into account the 2 nm silicon oxide layer on top of the silicon wafer which has a significantly lower thermal conductivity of 1.4 W/(mK). The base silicon wafer has a thermal conductivity of 34 W/(mK). The simulations are performed using the measured resistivity for the thin films i.e. W (300 $\mu\Omega$-cm), CoFeB (64 $\mu\Omega$-cm)

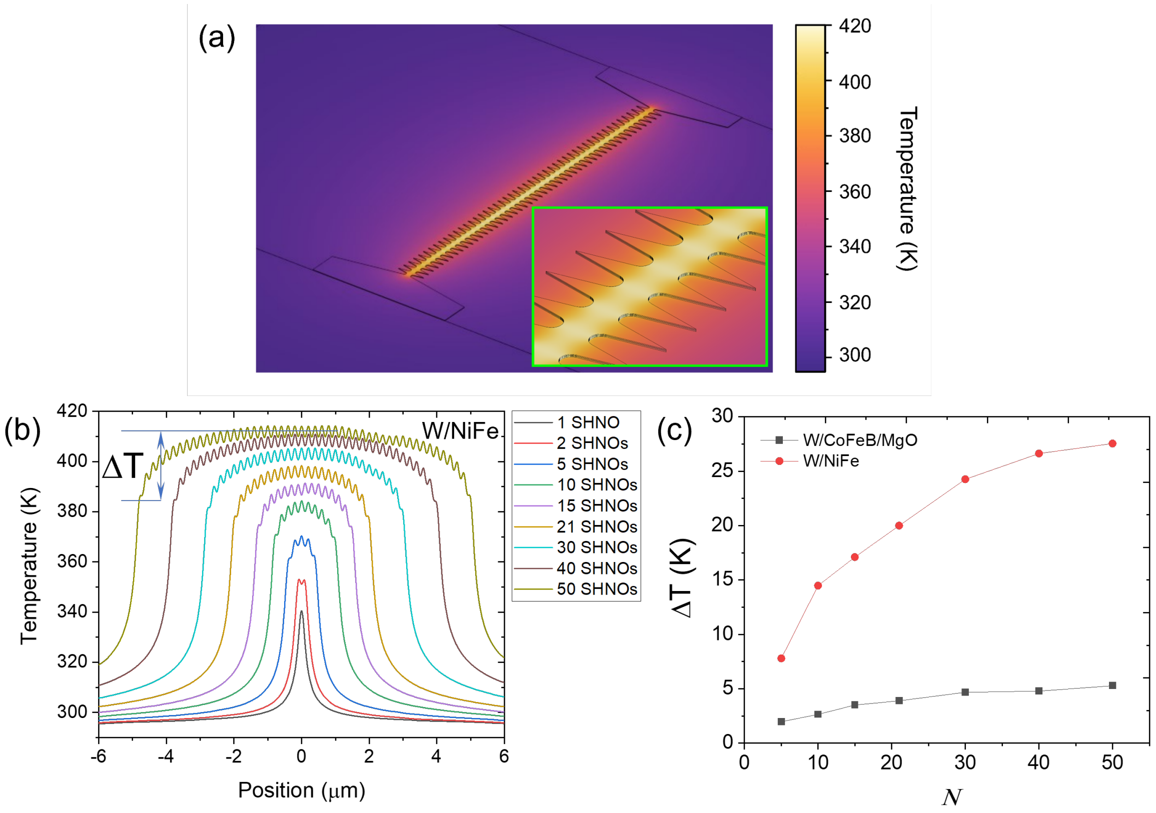


Figure S5: (a) Spacial map of temperature gradient in 50 serially connected nano-constriction geometry, inset shows the zoomed image of the center. (b) Simulated temperature for 1-50 NC-SHNOs of W/NiFe at $I_{DC}$ = 1.9 mA ($\Delta$T shows the temperature difference between centermost and outermost SHNOs). (c) $\Delta$T simulated for varying number of SHNOs for W(5 nm)/NiFe(3 nm)/$Al_2O_3$(4 nm) at $I_{DC}$ = 1.9 mA and W(5 nm)/CoFeB(1.4 nm)/MgO(2 nm)/$Al_2O_3$(4 nm) at $I_{DC}$ = 0.5 mA.

and NiFe (40 $\mu\Omega$-cm). In order to reduce the simulation time and resources we simulate a limited chip area of 1.5×1.5×0.5 mm. Temperature boundary conditions of 293.15 K are applied at the edges of the simulated area. Figure S5(a) shows a spacial thermal map for an applied DC current of 1.9 mA flowing through the chain of 50 NC-SHNOs with W/NiFe stack. In order to visualize the temperature gradients we have plotted a temperature profiles along x-axis for different number of constrictions in Figure S5(b). It can be seen that the temperature gradient exponentially increases to the edge of an array. In the Figure S5(c) we summarize the temperature difference between the central SHNOs and the ones on the edge for two stacks. In the case of W(5 nm)/NiFe(3 nm)/$Al_2O_3$(4 nm) stack at $I_{DC}$ = 1.9 mA the

temperature deviation start at 7.8 K for 5 NC-SHNOs and gradually increases to a 27.6 K. In the case of W(5 nm)/CoFeB(1.4 nm)/MgO(2 nm)/$Al_2O_3$(4 nm) stack we observe sufficiently smaller temperature deviation due to a smaller current of $I_{DC} = 0.5$ mA which together with the corresponding resistivity of the stacks results in 5.5 times lower Joule heating power.

## References


(1) Kittel, C. On the Theory of Ferromagnetic Resonance Absorption. Phys. Rev. **1948**, 73, 155–161.

(2) Behera, N.; Fulara, H.; Bainsla, L.; Kumar, A.; Zahedinejad, M.; Houshang, A.; Åkerman, J. Energy-Efficient $W_{100-x}Ta_x$/Co-Fe-B/MgO Spin Hall Nano-Oscillators. Phys. Rev. Appl. **2022**, 18, 024017.

(3) Liu, L.; Moriyama, T.; Ralph, D. C.; Buhrman, R. A. Spin-Torque Ferromagnetic Resonance Induced by the Spin Hall Effect. Phys. Rev. Lett. **2011**, 106, 036601.

(4) Dvornik, M.; Awad, A. A.; Åkerman, J. Origin of magnetization auto-oscillations in constriction-based spin Hall nano-oscillators. Phys. Rev. Appl. **2018**, 9, 014017.

(5) Houssameddine, D.; Florez, S.; Katine, J.; Michel, J.-P.; Ebels, U.; Mauri, D.; Ozatay, O.; Delaet, B.; Viala, B.; Folks, L.; Terris, B. D.; Cyrille, M.-C. Spin transfer induced coherent microwave emission with large power from nanoscale MgO tunnel junctions. Appl. Phys. Lett. **2008**, 93, 022505.

(6) Kubota, H.; Yakushiji, K.; Fukushima, A.; Tamaru, S.; Konoto, M.; Nozaki, T.; Ishibashi, S.; Saruya, T.; Yuasa, S.; Taniguchi, T.; Arai, H.; Imamura, H. Spin-torque oscillator based on magnetic tunnel junction with a perpendicularly magnetized free layer and in-plane magnetized polarizer. Appl. Phys. Exp. **2013**, 6, 103003.

(7) Zeng, Z.; Amiri, P. K.; Krivorotov, I. N.; Zhao, H.; Finocchio, G.; Wang, J.-P.; Katine, J. A.; Huai, Y.; Langer, J.; Galatsis, K.; Wang, K. L.; Jiang, H. High-power coherent microwave emission from magnetic tunnel junction nano-oscillators with perpendicular anisotropy. ACS Nano **2012**, 6, 6115–6121.

(8) Deac, A. M.; Fukushima, A.; Kubota, H.; Maehara, H.; Suzuki, Y.; Yuasa, S.; Nagamine, Y.; Tsunekawa, K.; Djayaprawira, D. D.; Watanabe, N. Bias-driven high-power microwave emission from MgO-based tunnel magnetoresistance devices. Nat. Phys. **2008**, 4, 803–809.

(9) Zeng, Z.; Upadhyaya, P.; Khalili Amiri, P.; Cheung, K.; Katine, J.; Langer, J.; Wang, K.; Jiang, H. Enhancement of microwave emission in magnetic tunnel junction oscillators through in-plane field orientation. Appl. Phys. Lett. **2011**, 99, 032503.

(10) Sharma, R.; Mishra, R.; Ngo, T.; Guo, Y.-X.; Fukami, S.; Sato, H.; Ohno, H.; Yang, H. Electrically connected spin-torque oscillators array for 2.4 GHz WiFi band transmission and energy harvesting. Nat. Commun. **2021**, 12, 1–10.

(11) Tsunegi, S.; Taniguchi, T.; Lebrun, R.; Yakushiji, K.; Cros, V.; Grollier, J.; Fukushima, A.; Yuasa, S.; Kubota, H. Scaling up electrically synchronized spin torque oscillator networks. Sci. Rep. **2018**, 8, 1–7.

(12) Costa, J. D.; Serrano-Guisan, S.; Lacoste, B.; Jenkins, A. S.; Böhnert, T.; Tarequzzaman, M.; Borme, J.; Deepak, F. L.; Paz, E.; Ventura, J.; Ferreira, R.; Freitas, P. P. High power and low critical current density spin transfer torque nano-oscillators using MgO barriers with intermediate thickness. Sci. Rep. **2017**, 7, 1–9.

(13) Seki, T.; Sakuraba, Y.; Arai, H.; Ueda, M.; Okura, R.; Imamura, H.; Takanashi, K. High power all-metal spin torque oscillator using full Heusler Co2 (Fe, Mn) Si. Appl. Phys. Lett. **2014**, 105, 092406.

(14) Lebrun, R.; Tsunegi, S.; Bortolotti, P.; Kubota, H.; Jenkins, A.; Romera, M.; Yakushiji, K.; Fukushima, A.; Grollier, J.; Yuasa, S., et al. Mutual synchronization of spin torque nano-oscillators through a long-range and tunable electrical coupling scheme. Nat. Commun. **2017**, 8, 1–7.

(15) Pribiag, V.; Krivorotov, I.; Fuchs, G.; Braganca, P.; Ozatay, O.; Sankey, J.; Ralph, D.; Buhrman, R. Magnetic vortex oscillator driven by dc spin-polarized current. Nat. Phys. **2007**, 3, 498–503.

(16) Tsunegi, S.; Kubota, H.; Yakushiji, K.; Konoto, M.; Tamaru, S.; Fukushima, A.; Arai, H.; Imamura, H.; Grimaldi, E.; Lebrun, R.; Grollier, J.; Cros, V.; Yuasa, S. High emission power and Q factor in spin torque vortex oscillator consisting of FeB free layer. Appl. Phys. Exp. **2014**, 7, 063009.

(17) Tsunegi, S.; Yakushiji, K.; Fukushima, A.; Yuasa, S.; Kubota, H. Microwave emission power exceeding 10 $\mu$ W in spin torque vortex oscillator. Appl. Phys. Lett. **2016**, 109, 252402.

(18) Dussaux, A.; Grimaldi, E.; Rache Salles, B.; Jenkins, A. S.; Khvalkovskiy, A. V.; Bortolotti, P.; Grollier, J.; Kubota, H.; Fukushima, A.; Yakushiji, K.; Yuasa, S.; Cros, V.; Fert, A. Large amplitude spin torque vortex oscillations at zero external field using a perpendicular spin polarizer. Appl. Phys. Lett. **2014**, 105, 022404.

(19) Dussaux, A.; Georges, B.; Grollier, J.; Cros, V.; Khvalkovskiy, A. V.; Fukushima, A.; Konoto, M.; Kubota, H.; Yakushiji, K.; Yuasa, S.; Zvezdin, K. A.; Ando, K.; Fert, A. Large microwave generation from current-driven magnetic vortex oscillators in magnetic tunnel junctions. Nat. Commun. **2010**, 1, 1–6.

(20) Rippard, W. H.; Pufall, M. R.; Kaka, S.; Silva, T. J.; Russek, S. E. Current-driven microwave dynamics in magnetic point contacts as a function of applied field angle. Phys. Rev. B **2004**, 70, 100406.

(21) Maehara, H.; Kubota, H.; Suzuki, Y.; Seki, T.; Nishimura, K.; Nagamine, Y.; Tsunekawa, K.; Fukushima, A.; Deac, A. M.; Ando, K.; Yuasa, S. Large Emission Power over 2 µW with High Q Factor Obtained from Nanocontact Magnetic-Tunnel-Junction-Based Spin Torque Oscillator. Appl. Phys. Exp. **2013**, 6, 113005.

(22) Maehara, H.; Kubota, H.; Suzuki, Y.; Seki, T.; Nishimura, K.; Nagamine, Y.; Tsunekawa, K.; Fukushima, A.; Arai, H.; Taniguchi, T.; Imamura, H.; Ando, K.; Yuasa, S. High Q factor over 3000 due to out-of-plane precession in nano-contact spin-torque oscillator based on magnetic tunnel junctions. Appl. Phys. Exp. **2014**, 7, 023003.

(23) Sani, S.; Persson, J.; Mohseni, S. M.; Pogoryelov, Y.; Muduli, P.; Eklund, A.; Malm, G.; Käll, M.; Dmitriev, A.; Åkerman, J. Mutually synchronized bottom-up multi-nanocontact spin–torque oscillators. Nat. Commun. **2013**, 4, 1–7.

(24) Houshang, A.; Iacocca, E.; Dürrenfeld, P.; Sani, S. R.; Åkerman, J.; Dumas, R. K. Spin-wave-beam driven synchronization of nanocontact spin-torque oscillators. Nat. Nano. **2016**, 11, 280–286.

(25) Duan, Z.; Smith, A.; Yang, L.; Youngblood, B.; Lindner, J.; Demidov, V. E.; Demokritov, S. O.; Krivorotov, I. N. Nanowire spin torque oscillator driven by spin orbit torques. Nat. Commun. **2014**, 5, 1–7.

(26) Awad, A. A.; Dürrenfeld, P.; Houshang, A.; Dvornik, M.; Iacocca, E.; Dumas, R. K.; Åkerman, J. Long-range mutual synchronization of spin Hall nano-oscillators. Nat. Phys. **2016**, 13, 292–299.

(27) Fulara, H.; Zahedinejad, M.; Khymyn, R.; Awad, A.; Muralidhar, S.; Dvornik, M.; Åkerman, J. Spin-orbit torque–driven propagating spin waves. Sci. Adv. **2019**, 5, eaax8467.

(28) Zahedinejad, M.; Awad, A. A.; Muralidhar, S.; Khymyn, R.; Fulara, H.; Mazraati, H.;

Dvornik, M.; Åkerman, J. Two-dimensional mutually synchronized spin Hall nano-oscillator arrays for neuromorphic computing. Nat. Nano. **2020**, 15, 47–52.

(29) Chen, J.-R.; Smith, A.; Montoya, E. A.; Lu, J. G.; Krivorotov, I. N. Spin–orbit torque nano-oscillator with giant magnetoresistance readout. Commun. Phys. **2020**, 3, 1–8.

(30) Demidov, V.; Urazhdin, S.; Zholud, A.; Sadovnikov, A.; Demokritov, S. Nanoconstriction-based spin-Hall nano-oscillator. Appl. Phys. Lett. **2014**, 105, 172410.

(31) Zahedinejad, M.; Mazraati, H.; Fulara, H.; Yue, J.; Jiang, S.; Awad, A.; Åkerman, J. CMOS compatible W/CoFeB/MgO spin Hall nano-oscillators with wide frequency tunability. Appl. Phys. Lett. **2018**, 112, 132404.
